# Nomophobia and Adolescent Social Isolation in a Rapidly Transforming Cultural Context: Evidence From Kyrgyzstan

**DOI:** 10.1002/puh2.70286

**Published:** 2026-06-01

**Authors:** Niyazi Ayhan

**Affiliations:** ^1^ Faculty of Communication Kyrgyz‐Turkish Manas University Bishkek Kyrgyzstan

**Keywords:** adolescents, digital behaviour, Kyrgyzstan, nomophobia, social isolation

## Abstract

This study investigates the association between nomophobia and social isolation among 465 adolescents aged 13–17 in Kyrgyzstan, a society experiencing rapid cultural and digital transformation. Digital dependence was operationalised using the Nomophobia Questionnaire, and social isolation was measured through a validated self‐report scale. Hierarchical regression analyses indicated that higher levels of nomophobia were significantly associated with greater social isolation (*β* = 0.31, *p* < 0.001), explaining approximately 10% of the variance in isolation scores after controlling for demographic variables. Gender, number of close friends and primary communication mode were also associated with social isolation, whereas age and school grade were not significant predictors. The findings suggest that nomophobia is meaningfully associated with adolescents’ perceived social connectedness in transitional cultural settings. Implications for school‐based prevention strategies and youth‐focused digital literacy interventions are discussed.

## Graphical abstract

This study examined the association between nomophobia and social isolation among 465 adolescents aged 13–17 in Kyrgyzstan. Higher nomophobia was significantly associated with greater social isolation, even after demographic variables were controlled for.

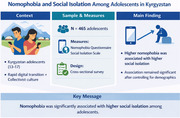

## 1 Introduction

Today, digitalisation has permeated nearly all areas of life. Smartphones now play a central role in identity formation and social connection, particularly for adolescents. Beyond communication, research shows that youth aged 13–17 often view smartphones as sources of psychological security and experience stress when separated from them [1, 2, 3]. According to the GSMA [4], young users are the most immersed demographic in mobile technology use.

This has given rise to nomophobia, a distinct construct referring to the fear, discomfort or anxiety experienced when individuals are unable to access their smartphone, remain connected or communicate through it and which includes cognitive, emotional and behavioural responses [5, 6]. Nomophobia is linked to anxiety, depression, and low self‐efficacy [2] and negatively impacts emotional stability and social connection [7]. Studies also indicate that increased smartphone use exacerbates nomophobia through loneliness and anxiety [8]. It is not merely fear of being offline but a multidimensional syndrome tied to psychological dependence and identity disruption. At the conceptual level, digital dependence, smartphone addiction and nomophobia should not be treated as interchangeable constructs, although they are closely related within the broader literature on problematic technology use. Digital dependence may be understood as an umbrella concept referring to persistent psychological or behavioural reliance on digital devices and online connectivity [9]. Smartphone addiction, by contrast, generally denotes excessive, compulsive and poorly regulated smartphone use resembling other forms of behavioural addiction [9, 10]. Nomophobia is more specific and refers to the distress associated with being unable to use one's mobile phone, communicate, access information or remain connected [6]. In this sense, nomophobia is best understood as a related but conceptually distinct manifestation of problematic digital engagement rather than a synonym for smartphone addiction. In the present study, digital dependence is used only as a broader conceptual backdrop. In contrast, nomophobia is treated as the primary analytical construct because the study's theoretical focus and measurement framework directly address fear of disconnection and mobile inaccessibility.

Recent scholarship suggests that nomophobia should be situated within a broader spectrum of problematic digital behaviour rather than being treated solely as anxiety about losing smartphone access. Within this broader perspective, digital overuse reflects not only time spent online but also dysregulated engagement, weakened self‐control and psychosocial strain. Özbay et al. [11] introduced the concept of digital obesity to describe excessive and dysfunctional forms of digital consumption, thereby expanding current discussions of digital dependence. In a related study, Özbay [12] reported that digital obesity was closely associated with digital addiction and lower desire for self‐control among university students. Complementing this line of work, Özbay [13] showed that prolonged immersion in low‐quality digital content may contribute to cognitive fatigue, reduced concentration, emotional blunting and social withdrawal. Although these studies were conducted with university students, they provide a broader conceptual basis for understanding digital overuse as a multidimensional behavioural condition with emotional, cognitive and social consequences. In this sense, nomophobia may be interpreted as one manifestation of maladaptive digital dependence, with potential implications for adolescents’ well‐being and their capacity to maintain meaningful offline relationships.

Adolescents are particularly vulnerable to nomophobia. It has been associated with attention problems, insomnia, poor academic performance and weakened interpersonal relationships [14, 15]. Many scholars see it as a form of psychosocial alienation, reinforcing social withdrawal and emotional disconnection.

This disconnection is often intensified by social isolation, defined as a decline in meaningful interaction, emotional support and peer relationships [16]. Adolescence—especially between ages 13 and 17—is a critical period marked by emotional, cognitive and social change [17, 18]. Young people's responses to digital environments can vary significantly across early (13–14), middle (15–16) and late adolescence (17).

Paradoxically, although smartphones are often used to cope with loneliness, their overuse can worsen isolation. More broadly, problematic digital engagement may undermine real‐world relationships and emotional regulation, whereas nomophobia represents one specific form through which this vulnerability may become psychologically salient [19, 20]. For example, social media addiction has been shown to erode adolescents’ capacity for emotional bonding.

Recent research shows that nomophobia correlates with unmet psychological needs and insecure attachment styles, especially in girls [7, 21]. Digital communication often fails to replace authentic social bonds [19], and the relationship between nomophobia and social isolation appears deeply intertwined.

Despite growing international attention to nomophobia, studies directly linking it to social isolation have been concentrated largely in settings where patterns of digital individualisation are already well established. Much less is known about how this association operates in societies undergoing rapid digital expansion while still maintaining strong collectivist norms, dense family structures and face‐to‐face relational expectations. Kyrgyzstan offers an analytically important context in this respect. As a society shaped by post‐Soviet transformation, accelerated smartphone penetration and enduring collectivist social values, it provides an opportunity to examine whether the nomophobia–social isolation association remains meaningful under conditions where adolescents are socialised simultaneously into family‐based interdependence and increasingly individualised digital practices [22]. This concern is also consistent with recent evidence from Kyrgyzstan showing that problematic digital engagement is associated with loneliness among young adults, suggesting that the psychosocial consequences of excessive digital use may extend across different age groups in the country [23]. Accordingly, the present study does not merely replicate an established association in a new location; rather, it examines whether a widely discussed digital‐age psychosocial pattern also emerges in a culturally transitional setting and how that pattern should be interpreted within the specific developmental and sociocultural conditions of adolescence in Kyrgyzstan.

### 1.1 Research Questions and Hypotheses

This study is guided by the following research questions and hypotheses, with particular attention to the developmental and cultural context of adolescents in Kyrgyzstan—a society undergoing rapid digital transformation within a traditionally collectivist structure.

RQ1. What are the levels of nomophobia among adolescents aged 13–17 in Kyrgyzstan?

Nomophobia, defined as the anxiety or distress associated with being unable to access or use one's mobile phone, has been shown to disrupt emotional regulation and social connectedness during adolescence*. However, empirical data from Central Asian contexts, particularly Kyrgyzstan, remain limited* [1, 2].

RQ2. What are the levels of social isolation among adolescents aged 13–17 in Kyrgyzstan?

*Adolescence is a formative period for social belonging and identity construction. Increased exposure to digital screens and reduced in‐person interaction may increase the risk of social isolation in this age group* [24].

RQ3. Do levels of nomophobia and social isolation differ significantly according to demographic variables such as gender, age and daily phone usage time?

*Previous studies suggest that psychosocial risks, such as nomophobia and social isolation, vary by demographic factors. For instance, higher nomophobia prevalence has been reported among females, and factors such as age and daily screen time may play significant roles in shaping these experiences* [7, 8, 25].

RQ4. Does nomophobia significantly predict levels of social isolation among adolescents?

H_1_: Nomophobia is a significant predictor of social isolation.

RQ5. Is there a statistically significant relationship between nomophobia and social isolation levels among adolescents?

H_2_: There is a positive and significant correlation between nomophobia and social isolation among adolescents.

## 2 Methodology

### 2.1 Research Design

This study employed a correlational survey model, a widely accepted quantitative research design used to identify the level and direction of relationships between variables without implying causal inference [26]. This approach was deemed appropriate to explore the interaction between psychosocial constructs such as nomophobia and social isolation, particularly during adolescence (ages 13–17)—a critical developmental period characterised by heightened sensitivity to social connection and technological exposure.

Considering Kyrgyzstan's unique blend of traditional values and rapid digital change, this study was designed to capture adolescents’ digital experiences in their real cultural context. Data were collected directly from students using well‐established measurement tools, and multiple regressions were used to examine which factors might predict specific outcomes. This method aligns with best practices in social science research, which emphasise studying people in their everyday environments so that results accurately reflect their real‐life experiences [27].

### 2.2 Participants, Sampling Procedures and Ethical Considerations

The data for this study were collected in February 2025 from adolescents aged 13–17 living in Bishkek, the capital of Kyrgyzstan. Participants were selected using simple random sampling, and participation in the study was entirely voluntary. No formal a priori power analysis was conducted; however, the final sample size of 465 was considered adequate for the planned analyses. In regression‐based research, sample size recommendations commonly suggest at least 50 + 8 m cases for testing multiple predictors, where m refers to the number of predictors included in the model [28]. Given the number of variables included in the hierarchical regression models, the present sample exceeded the recommended threshold. In addition, the achieved sample size was considered sufficient for confirmatory factor analysis (CFA) and other multivariate procedures, which generally require relatively large samples to obtain stable parameter estimates [29]. Therefore, the final sample was considered methodologically adequate for the analytical strategy adopted in this study. Data were collected through face‐to‐face paper‐based surveys.

Survey forms were distributed to participants through their families, and data were collected only from adolescents who used smartphones. Participants and their families provided informed consent by ticking the consent box on the printed form. The consent form clearly stated the study's purpose, the voluntary nature of participation and the principle of confidentiality.

The research was conducted in accordance with the World Medical Association's Declaration of Helsinki [30] to protect participants’ rights and ensure compliance with ethical principles. The sampling method used was simple random sampling, which is often recommended to reduce bias and increase representativeness [27, 31].

### 2.3 Measurement

The data collection tool used in this study consists of four sections:

(1) Demographic information, (2) digital device usage habits, (3) nomophobia scale and (4) social isolation scale (SIS).

The first section includes questions about participants’ gender, age, grade level, smartphone ownership, phone usage time (daily and annual), preferred social media platforms and usage frequency. This section assessed participants’ digital behaviour patterns and used them as control variables in the analyses.

### 2.4 The Nomophobia Questionnaire (NMP‐Q)

Originally developed by Yildirim and Correia [6], it was used to assess adolescents’ levels of nomophobia. The original scale consists of 20 items and a four‐factor structure reflecting not being able to access information, giving up convenience, not being able to communicate and losing connectedness. Higher scores indicate higher levels of nomophobia.

For the present study, the scale was linguistically and culturally adapted for the Russian‐speaking adolescent sample in Kyrgyzstan. The adaptation process involved bilingual experts and field specialists to ensure linguistic clarity, conceptual appropriateness and cultural relevance. Permission to use and adapt the scale was obtained from the scale developers. In the adapted version, the response format was arranged as a 5‐point Likert scale (1 = Never, 5 = Always). A pilot application was conducted with 25 participants to evaluate item clarity and comprehensibility before the main data collection.

To examine whether the adapted version preserved the original factor structure, a CFA was conducted. Consistent with the original scale, a four‐factor model was tested in R using the lavaan package. Because the item responses were treated as ordinal, the weighted least squares mean and variance adjusted (WLSMV) estimator was used. The CFA results generally supported the original four‐factor structure of the scale, yielding acceptable‐to‐good fit indices, *χ* ^2^(164) = 548.41, CFI = 0.982, TLI = 0.979, RMSEA = 0.071 and SRMR = 0.070. Standardised factor loadings ranged from 0.564 to 0.890, and all loadings were statistically significant. In the original study, Cronbach's *α* was reported as 0.9369; in the present study, the internal consistency coefficient was Cronbach's *α *= 0.884.

### 2.5 Social Isolation Scale

The SIS, developed by Çelikbaş and Tatar [32], was used to assess adolescents’ levels of social isolation. The original scale consists of 14 items and was designed to measure social isolation as a unidimensional construct. Higher scores indicate higher levels of perceived social isolation.

For the present study, the scale was adapted for use with the Russian‐speaking adolescent sample in Kyrgyzstan. The adaptation process involved bilingual experts and field specialists to ensure linguistic clarity, conceptual appropriateness and cultural relevance. Permission to use and adapt the scale was obtained from the scale developers. In the adapted version, the response format was arranged as a 5‐point Likert scale (1 = Never, 5 = Always). A pilot application was conducted to evaluate the comprehensibility and contextual suitability of the items before the main data collection.

To examine whether the adapted version preserved its intended structure, a CFA was conducted in R using the lavaan package. Because the item responses were treated as ordinal, the WLSMV adjusted estimator was used. The CFA results generally supported the unidimensional structure of the scale, yielding acceptable overall model fit, *χ* ^2^(77) = 323.88, CFI = 0.990, TLI = 0.988, RMSEA = 0.083 and SRMR = 0.065. Standardised factor loadings ranged from 0.640 to 0.884, and all factor loadings were statistically significant. In the present study, the internal consistency coefficient of the scale was Cronbach's *α* = 0.921.

### 2.6 Data Analysis

Data analysis was conducted using IBM SPSS Statistics 23.0 and R. Descriptive statistics, including frequencies, percentages, means and standard deviations, were used to summarise participants’ demographic characteristics and smartphone use behaviours. Before the primary analyses, the internal consistency of the scales was assessed using Cronbach's *α* coefficients. The NMP‐Q demonstrated high internal consistency (*α* = 0.884), and the SIS also showed excellent reliability (*α* = 0.921) [33].

In addition, CFA was performed for both adapted scales in R using the lavaan package in order to examine whether their original factor structures were retained in the present sample. Because the item responses were treated as ordinal, the WLSMV adjusted estimator was used. The original four‐factor structure of the NMP‐Q was tested. The CFA results generally supported this model, with acceptable‐to‐good fit indices, *χ* ^2^(164) = 548.41, CFI = 0.982, TLI = 0.979, RMSEA = 0.071 and SRMR = 0.070. The original unidimensional structure of the SIS was tested. The CFA results also supported this model, yielding acceptable overall fit, *χ* ^2^(77) = 323.88, CFI = 0.990, TLI = 0.988, RMSEA = 0.083 and SRMR = 0.065. Standardised factor loadings for both scales were statistically significant.

The normality of the distributions was assessed using the Kolmogorov–Smirnov and Shapiro–Wilk tests. Although both tests yielded statistically significant results (*p* < 0.05), the large sample size (*N* = 465) and visual inspection of histograms and *Q*–*Q* plots indicated approximate normality [34], supporting the use of parametric analyses. Following the assessment of assumptions, Pearson correlation analysis was used to examine the strength and direction of the relationship between nomophobia and social isolation. In addition, two‐step hierarchical regression and simple linear regression analyses were conducted to examine whether nomophobia was statistically associated with social isolation, after relevant demographic and digital‐use variables were controlled for. Before conducting the regression analyses, the main assumptions of regression were examined. Linearity and homoscedasticity were assessed through visual inspection of scatterplots and standardised residual plots. Independence of errors was evaluated using the Durbin–Watson statistic. Multicollinearity was examined using tolerance and variance inflation factor (VIF) values. Although age and grade level showed relatively elevated VIF values, the results did not indicate critical multicollinearity, and these variables were interpreted with caution. All analyses were conducted at a 95% confidence level, with statistical significance set at *p* < 0.05.

### 2.7 Demographics and Digital Media Use Among Adolescents

Table 1 shows the demographic distribution and digital media usage habits of the 465 adolescent participants in the study. 51.2% of the participants were female, and 48.8% were male. The age distribution is evenly distributed between 13 and 17 years. All age groups are represented similarly, with the highest proportion (21.3%) in the 13‐year‐old group. This allows for reliable comparisons of age‐related behavioural and psychological variables.

**TABLE 1:** Demographics and digital media use among adolescents (*N* = 465).

Variable	Category	*N*	Percentage
**Gender**	Girl	238	51.2
	Boy	227	48.8
	Total	465	100.0
**Age**	13	99	21.3
	14	90	19.4
	15	95	20.4
	16	91	19.6
	17	90	19.4
	Total	465	100.0
**Grade**	7	163	35.1
	8	31	6.7
	9	78	16.8
	10	106	22.8
	11	87	18.7
	Total	465	100.0
**Duration of smartphone use (years)**	1 year or less	26	5.6
	2–3 years	58	12.5
	4–5 years	117	25.2
	+5	264	56.8
	Total	465	100.0
**Daily smartphone use duration (h)**	Less than 1 h	13	2.8
	1–3 h	127	27.3
	4–6 h	194	41.7
	7+	131	28.2
	Total	465	100.0

The distribution by grade level was as follows: 7th‐grade students (35.1%), 8th‐grade students (6.7%), 9th‐grade students (16.8%), 10th‐grade students (22.8%) and 11th‐grade students (18.7%).

Regarding smartphone usage duration, 56.8% of participants have owned a smartphone for over 5 years. This high percentage indicates that young individuals have been exposed to digital devices from an early age. The usage rate between 4 and 5 years is also noteworthy (25.2%).

In terms of daily phone usage time, 41.7% of the sample uses their phones for 4–6 h, whereas 28.2% use them for more than 7 h. When these two categories are combined, approximately 70% of participants are heavy daily smartphone users.

Table 2 shows how adolescents use smartphones, which platforms they prefer to communicate on, and how often they participate in social activities. Most participants (59.4%) said their families warned them about online risks but did not set any limits. This points to a relatively unrestricted digital environment where personal responsibility becomes more central. Meanwhile, 23% of families did not intervene at all, and 7.5% said these topics never came up.

**TABLE 2:** Self‐reported smartphone use, online behaviour and psychosocial engagement among adolescents (*N* = 465).

Variable	Category	*N*	Percentage
Family attitudes toward internet and social media use	They actively monitor and restrict usage	47	10.1
	They warn about risks but do not restrict	276	59.4
	They do not intervene	107	23.0
	We never talk about these topics	35	7.5
Primary communication platform with friends	Face‐to‐face	339	72.9
	WhatsApp	70	15.1
	Instagram	43	9.2
	Discord	12	2.6
	Telegram	1	0.2
Participation in social activities	Always participate	149	32.0
	Sometimes participate	227	48.8
	Never participate	89	19.1
Emotional response when a smartphone is not present	I feel calm	193	41.5
	I feel slightly anxious	165	35.5
	I feel anxious	74	15.9
	I panic	33	7.1
Number of close friends	None	18	3.9
	1–2 friends	37	8.0
	3–5 friends	123	26.5
	6 or more friends	287	61.7
Time to notice smartphone absence	Immediately notice	206	44.3
	Within 10–30 min	221	47.5
	Within 1–2 h	30	6.5
	After a longer time	8	1.7
Purpose of smartphone use	Social media	407	87.5
	News/Discussion platforms	115	24.7
	Entertainment	306	65.8
	Education	257	55.3

*Note:* Participants were allowed to select more than one option for smartphone use purposes. The table presents the number and percentage of respondents who answered ‘Yes’ to each option.

When adolescents’ communication preferences with their friends are examined, face‐to‐face communication is still dominant, with a rate of 72.9%. However, digital platforms (WhatsApp 15.1%, Instagram 9.2%) are also important alternatives.

Participants’ level of participation in social activities is also striking: 48.8% sometimes and 32% always participate in social activities. On the other hand, 19.1% reported not participating in any social activities. In studies related to social isolation, such data are important for identifying at‐risk groups.

When the emotional reactions when the smartphone was not with them were evaluated, 41.5% reported feeling comfortable, 35.5% reported mild anxiety, 15.9% reported overt anxiety, and a small group of 7.1% reported panic. These findings suggest that nomophobia levels vary and that emotional reactions should be examined in the context of individual differences.

Regarding the number of close friends, 61.7% of the participants reported six or more friends. At the same time, this indicates the breadth of the social circle; it requires a multidimensional analysis of other psychosocial variables related to feelings of loneliness. About 44.3% of participants said their phone was missing immediately, and 47.5% became aware within 10–30 min. Such quick reactions suggest a strong habitual focus on the device and may reflect heightened sensitivity to smartphone absence, which is conceptually consistent with nomophobia. Finally, regarding the purpose of smartphone use, 87.5% of participants reported preferring social media, 65.8% entertainment, 55.3% education and 24.7% news and discussion platforms. These data, which allow more than one option, reveal the multifunctional use of digital devices and that users respond to different digital needs.

These findings provide important context for understanding the nomophobia and social isolation variables and will serve as the basis for statistical analyses.

### 2.8 Predicting Social Isolation With Nomophobia and Demographic Variables: Hierarchical Regression Findings

A two‐step hierarchical regression analysis was conducted to examine the variables associated with social isolation (Table 3). Demographic characteristics and digital usage patterns were included as control variables in Model 1. The model was consistently significant, explaining 18.5% of the variance in social isolation (*R* ^2^ = 0.185; *F*(9,454) = 4.650; *p* < 0.001). The variables found to be significant in this model were gender (*β* = 0.152; *p* = 0.001), environment used for communication with friends (*β* = 0.144; *p* = 0.002) and number of close friends (*β* = −0.155; *p* = 0.001). According to this separation, women exhibited higher levels of social isolation. In contrast, individuals who communicated face‐to‐face with others remained less isolated, with limitations observed in the isolation levels of the number of close friends.

**TABLE 3:** Hierarchical regression analysis predicting social isolation.

Predictors	*B*	SE *B*	*β*	*t*	*p*	Tolerance	VIF	Model
Gender	0.234	0.071	0.152	3.315	0.001	0.957	1.045	**Model 1**
Age	−0.086	0.065	−0.159	−1.328	0.185	0.141	7.116	
Grade	0.095	0.06	0.192	1.589	0.113	0.137	7.273	
Years using smartphone	0.019	0.041	0.022	0.461	0.645	0.864	1.158	
Daily smartphone use	0.033	0.046	0.035	0.713	0.476	0.854	1.172	
Family attitude towards tech	−0.032	0.047	−0.031	−0.679	0.498	0.953	1.049	
Primary communication platform	0.141	0.045	0.144	3.13	0.002	0.956	1.046	
Participation in social activities	−0.034	0.05	−0.031	−0.676	0.499	0.968	1.033	
Number of close friends	−0.149	0.044	−0.155	−3.341	0.001	0.939	1.065	
Gender	0.087	0.073	0.056	1.19	0.235	0.839	1.192	**Model 2**
Age	−0.096	0.063	−0.178	−1.534	0.126	0.14	7.122	
Grade	0.109	0.058	0.222	1.891	0.059	0.137	7.286	
Years using smartphone	0.003	0.04	0.004	0.081	0.935	0.86	1.163	
Daily smartphone use	−0.012	0.045	−0.013	−0.275	0.783	0.828	1.208	
Family attitude towards tech	−0.019	0.046	−0.019	−0.417	0.677	0.951	1.052	
Primary communication platform	0.12	0.044	0.123	2.751	0.006	0.949	1.054	
Participation in social activities	−0.007	0.048	−0.007	−0.152	0.879	0.959	1.042	
Number of close friends	−0.14	0.043	−0.146	−3.254	0.001	0.938	1.066	
Nomophobia (NF_Total)	0.266	0.046	0.277	5.756	0.0	0.815	1.227	

Abbreviation: VIF, variance inflation factor.

In Model 2, the total nomophobia score was added. This resulted in a statistically significant increase in explained variance (Δ*R* ^2^ = 0.068), with the full model accounting for 26.3% of the variance in social isolation (*R* ^2^ = 0.263; *F*(10,453) = 7.794; *p* < 0.001). Nomophobia was positively and significantly associated with social isolation (*β* = 0.277; *p* < 0.001). Although this increase indicates that nomophobia contributed meaningfully to the model beyond the control variables, the explained variance should be interpreted cautiously, as the cross‐sectional design does not allow causal inference. Furthermore, a simple linear regression analysis showed that nomophobia was significantly associated with social isolation (*F*(1,463) = 49.18; *p* < 0.001), explaining 9.6% of the variance (*R* ^2^ = 0.096). Although this level of explained variance is modest, it still suggests that nomophobia represents a statistically relevant correlate of adolescents’ social isolation. However, it is clearly not the only factor involved. These findings indicate a statistically meaningful association, but not a causal one.

All these findings support our hypothesis: H_1_ Nomophobia is a significant predictor of social isolation.

### 2.9 Relationship Between Nomophobia and Social Isolation: Correlation and Regression Analyses

Pearson's correlation analysis revealed a statistically significant, positive relationship between nomophobia and social isolation (*r* = 0.310, *p* < 0.01; see Table 4). This finding indicates a significant positive association between nomophobia and social isolation, supporting hypothesis H_2_. Simple linear regression analysis showed that nomophobia was significantly and positively associated with social isolation (*β* = 0.31, *p* < 0.001); however, as this is a correlational study, this should not be interpreted as evidence of causality, and the nomophobia variable explained approximately 9.6% of the variance in social isolation (*R* ^2^ = 0.096). These results reveal that the H_2_ hypothesis is also supported.

**TABLE 4:** Correlation and regression analysis predicting social isolation from nomophobia (*N* = 465).

Analysis	Variable	*B*	SE	*β*	*t*	*p*	95% CI [LL, UL]
Pearson correlation	Nomophobia—social isolation	—	—	0.310	—	<0.01	—
Linear regression	Constant	1.005	0.116	—	8.671	<0.001	[0.777, 1.232]
	Nomophobia	0.298	0.042	0.310	7.012	<0.001	[0.214, 0.381]

## 3 Discussion

This study set out to explore how digitalisation is shaping the psychological and social lives of young people by looking at the links between nomophobia and social isolation among adolescents aged 13–17. The results showed clear differences across both demographic factors and digital habits. It is important to interpret these findings within the unique context of Kyrgyzstan. In this country, rapid cultural change means that traditional collectivist values are increasingly blending with more individualistic, digital lifestyles. In environments like this, a heavy reliance on digital technology can sharpen the clash between traditional family or community expectations and the new freedoms of the digital era, creating additional psychological and social challenges for young people [22]. This cultural context is particularly important for interpreting the present findings. In Kyrgyzstan, adolescents are growing up in a social environment where family cohesion, interdependence and respect for collective norms continue to shape everyday life, even as digital technologies expand individual autonomy and private forms of communication. Within such a setting, smartphone use may serve not only as a practical communication tool but also as a semiprivate space where adolescents negotiate identity, peer belonging and emotional independence. At the same time, this digital transition may intensify tension between offline relational obligations and online forms of self‐expression. For some adolescents, this tension may make constant digital connectedness feel psychologically necessary while also weakening direct forms of social engagement that are traditionally reinforced through family and face‐to‐face interaction. From this perspective, nomophobia and social isolation should be understood not only as individual‐level psychosocial outcomes but also as experiences shaped by the broader interaction between collectivist social expectations, family structure and rapid digital transformation in contemporary Kyrgyzstan [17, 18, 22].

These results are in line with previous research from different cultures, which has found that both gender and the ways adolescents use their smartphones can play a significant role in their risk for social isolation and problematic smartphone‐related distress [19, 35, 36]. Other studies have also shown that the effects of digital behaviours—and their impact on mental and social well‐being—can vary across cultural contexts. For adolescents in societies undergoing rapid transition, digitalisation may lead to stronger identity conflicts and greater family tension than in more stable cultural environments [17, 18, 22].

Our findings show that girls report higher levels of nomophobia than boys (see Table A1). This is not surprising—other studies have found similar results. For example, Beyens et al. [35] noted that women are more likely to get emotionally attached through social media, which can make them more vulnerable to problematic smartphone engagement and nomophobia‐related distress. Elhai et al. [36] also found that girls tend to react more emotionally and feel more anxious about using smartphones. Studies from Central Asia confirm that these gender differences are not unique—they also appear in various cultural settings. These studies suggest that cultural factors can increase technology‐related anxiety and intensify nomophobia‐related vulnerability in adolescent girls [7, 22].

No significant difference was observed based on age. This may be due to Generation Z's direct exposure to digital technologies. Keles et al. [16] also suggest that digitalisation is internalised homogeneously in this age group and that age‐related variance decreases. Research in developmental psychology also points out that adolescence is not the same experience for everyone, and the effects of digital habits on mental and social well‐being can vary depending on whether someone is in early, middle or late adolescence [17, 18]. How long someone spends on their smartphone each day also seems to matter—those who use their phones for more than 7 h a day tend to have much higher levels of nomophobia. This supports what Panova and Lleras [37] found: Spending much time on screens is not just a habit but can also be a sign of psychological dependence. The duration of participants’ awareness of their phone's absence and their emotional reactions also provides important psychological clues. In particular, individuals who ‘immediately notice’ or ‘panic’ have significantly higher levels of nomophobia. This result suggests that nomophobia may be associated not only with technological deprivation but also with attachment anxiety and loss of control [18, 38]. Similar results have been reported in studies from around the world—when teenagers get attached to their phones, they tend to feel anxious if they are separated from them, sometimes lose control over their usage and often find it challenging to manage their emotions [2, 6].

Social isolation findings revealed notable differences in terms of demographic and social variables (Table A2). Female participants had significantly higher levels of social isolation compared to males. Loades et al. [39] and Marques de Miranda et al. [40] also indicate that adolescent girls were more susceptible to loneliness and isolation during the pandemic. This may be due to levels of emotional expectation and social connectedness associated with gender roles [41]. Similar trends have been reported in multi‐country studies, which indicate that adolescent girls are more prone to loneliness and social isolation due to social role expectations and increased emotional sensitivity [40, 42].

We observed that social isolation was higher among individuals with fewer close friends. This matches what Hawkley and Cacioppo [43] found: When people feel less supported by their social circle, they are more likely to feel alone. The way participants communicated with friends—such as the app or platform they used—also made a real difference in how isolated they felt. The research also showed that the way participants kept in touch with their friends—that is, the communication platforms they used—played a significant role. Although levels of social isolation were significantly lower in individuals who preferred face‐to‐face communication, isolation was higher in those who communicated via social media platforms. This was also noted by Primack et al. [44] and Twenge et al. [45], who stated that visually oriented social media applications, in particular, increased feelings of social comparison and loneliness. Likewise, studies from other countries have found that choosing face‐to‐face conversations helps protect against feeling isolated, while relying mainly on online communication—primarily through image‐heavy social media—can make people feel more excluded and lonely [44, 45]. The study found that factors such as age, grade level, involvement in social activities, or family opinions about technology did not affect participants’ feelings of social isolation. This aligns with the view of Heinrich and Gullone [46], who argued that loneliness and social isolation are primarily tied to how much support and connection someone feels, rather than to their environment. In other words, social isolation among adolescents is less about outside factors and more about the quality of their relationships and whether they feel supported. This fits well with earlier psychosocial models as well [46].

One of the most notable findings of the present study is that adolescents who reported higher levels of nomophobia also tended to report higher levels of social isolation. This pattern indicates a statistically meaningful association between the two constructs during adolescence, rather than a directional causal relationship [6, 47].

The emergence of similar findings across different cultural contexts suggests that this association may be observed relatively consistently in adolescent populations. This pattern is also in line with recent studies conducted in other transitional societies, where technology‐related anxiety has been found to co‐occur with greater social withdrawal and emotional loneliness in adolescents [21, 25].

In this study, nomophobia was moderately and positively associated with social isolation. Adolescents with higher nomophobia scores also tended to report higher levels of isolation. Even after relevant demographic and digital‐use variables were controlled for, nomophobia remained significantly associated with social isolation (*β* = 0.310, *p* < 0.001; see Tables 3 and 4). However, this result should be interpreted as an association observed within a cross‐sectional design rather than as evidence of a directional causal effect. In the present model, nomophobia showed a stronger statistical association with social isolation than several of the demographic variables. At the same time, the model's explanatory power should be interpreted with caution. Although nomophobia was significantly associated with social isolation, the amount of variance explained in the simple regression model was modest, indicating that adolescents’ social isolation is likely shaped by a broader constellation of psychological, relational and contextual factors. In this sense, the present findings should be understood as identifying a meaningful correlate rather than offering a comprehensive explanatory model. Future research would benefit from testing more theoretically elaborated models that incorporate potential mediating and moderating mechanisms, such as loneliness, emotional regulation, peer relationship quality, family functioning or social anxiety, in order to clarify how and under what conditions nomophobia is linked to social isolation.

These findings align with previous research. For instance, Gezgin et al. [47] found that nomophobia is closely linked to loneliness and social withdrawal among young people. Recent studies from countries, such as Jordan and Kazakhstan, have reported similar patterns, showing that nomophobia is widespread among adolescents and is associated with poorer emotional balance, weaker social ties and higher stress levels [7, 22]. In contexts such as Kyrgyzstan, where traditional family values, interdependence and rapidly expanding digital freedoms coexist, these associations may become more visible because adolescents must navigate both collective expectations and increasingly individualised forms of digital life.

While Yıldırım and Correia [6] defined nomophobia as a form of ‘technological loneliness’ that leads individuals to withdraw from offline interactions, Elhai et al. [10] noted that nomophobia has direct effects on loneliness, as well as on social anxiety and depression. Therefore, the present findings suggest that explaining social isolation solely through environmental or demographic factors may be insufficient, as nomophobia also appears to be meaningfully associated with adolescents’ social experiences. A specific digital‐behaviour construct, such as nomophobia, appears to be significantly related to an individual's social relationships. This is especially relevant for adolescents, as greater reliance on smartphone‐based interaction may coincide with reduced face‐to‐face engagement and higher perceived isolation [48]. However, given the correlational nature of the present findings, the practical implications should be interpreted with caution. Rather than supporting direct intervention effects, the results point to the potential value of school‐ and family‐based awareness efforts focused on healthy technology habits, digital literacy and face‐to‐face social engagement. In rapidly changing contexts such as Kyrgyzstan, these approaches may be considered potential areas for preventive attention and future evidence‐based intervention development, rather than as outcomes directly established by the present study [20, 48].

Beyond the immediate findings, the present study also contributes to broader scholarship by showing that the association between nomophobia and social isolation should not be treated as a pattern limited to highly individualised or Western‐centred digital settings. The Kyrgyz case suggests that this relationship may also be salient in culturally transitional societies where adolescents remain embedded in collectivist family structures while increasingly relying on smartphones for privacy, autonomy and peer connection. In this sense, the study extends the literature by positioning nomophobia not simply as an outcome of excessive device use but as a psychosocial indicator whose meaning may be shaped by the tension between traditional relational norms and rapidly changing digital routines. The contribution of the study, therefore, lies not only in documenting a statistically significant association but also in showing why that association warrants interpretation within a broader sociocultural framework rather than as a context‐free behavioural pattern.

## 4 Conclusion

This study examined the relationship between nomophobia and social isolation among adolescents aged 13–17. The results showed a clear and positive association between the two variables: Higher nomophobia scores were associated with higher levels of social isolation (*r* = 0.310, *p* < 0.01). In the simple linear regression analysis, nomophobia was significantly associated with social isolation and accounted for approximately 9.6% of the variance in isolation scores (*β* = 0.310, *p* < 0.001). At a broader level, the findings suggest that this association is also relevant in culturally transitional settings such as Kyrgyzstan, where collectivist expectations and rapidly expanding digital practices coexist. However, because the model's explanatory power remained modest, nomophobia should be interpreted as a relevant correlate within a broader psychosocial network rather than as a sufficient standalone explanation of adolescent social isolation. The hierarchical regression analysis was consistent with this pattern. In the first model, demographic and digital behaviour variables accounted for 18.5% of the variance in social isolation. When nomophobia was added in the second model, the explained variance increased to 26.3%. This indicates that nomophobia contributed meaningfully to the model beyond the control variables; however, this contribution should be interpreted as a statistical association rather than as evidence of an independent causal effect.

The study also showed that factors, such as gender, the number of close friends and the way teens communicate, were closely linked to social isolation. Teens without close friends felt more isolated, and those who mainly used digital platforms like Instagram or Discord—rather than talking face‐to‐face—had higher levels of isolation. By contrast, factors, such as age, grade, joining activities or family perceptions of digital media, did not seem to make much difference. This means that whether teens feel primarily isolated depends on how they use digital technology and how close they feel to people around them, rather than their environment or family situation.

These results highlight the challenges adolescents may face in balancing their emotional and social needs in a technology‐shaped environment. Nomophobia, in particular, emerged as an important correlate of adolescents’ emotional and social difficulties, as it was associated with lower well‐being, weaker social connectedness and greater feelings of isolation in the present findings. Although the correlational design does not justify strong intervention claims, the findings may still have practical relevance for families, schools and youth professionals. In particular, they suggest that greater attention to healthy technology habits, face‐to‐face communication and digital literacy may be useful when designing preventive or supportive strategies for adolescents [16, 46]. Future studies should evaluate these approaches directly before drawing firm conclusions about their effectiveness in reducing social isolation or nomophobia‐related distress.

Because the present study is correlational, these implications should be understood as tentative and hypothesis‐generating rather than as evidence for the effectiveness of specific interventions.

### 4.1 Limitations and Future Research

This study provides valuable insight, but some limitations remain. Because the data were collected at a single point in time, it is impossible to say whether nomophobia causes social isolation or vice versa. Future research that tracks changes over time or uses experimental methods could help clarify how the two are connected. Another limitation concerns the possibility of bidirectional relationships and omitted‐variable bias. Although nomophobia was significantly associated with social isolation, the cross‐sectional design does not allow us to determine whether nomophobia is linked to later isolation, whether socially isolated adolescents become more dependent on smartphone‐based connection, or whether both are shaped by other psychosocial factors. In addition, variables such as depressive symptoms, social anxiety, family functioning and peer relationship quality were not included in the present models and may partly account for the observed association.

Second, the sample consists only of adolescents aged 13–17 and is limited to a specific geographical region. This may limit the generalisability of the findings. Comparative research across different age groups, sociocultural contexts and educational types may yield more holistic results. In particular, it should be considered that experiences of nomophobia and social isolation may differ between rural and urban areas.

Third, the study's data were collected through self‐reporting. This may affect data accuracy due to factors such as social favourability bias and cognitive distortions.

It is recommended that future studies use multiple data sources, such as observation, focus group interviews or parent/teacher reports.

Finally, this study's nomophobia and SISs have certain limitations in terms of contextual validity. In future studies, including broader psychosocial variables such as problematic digital behaviour, digital anxiety, loneliness, emotional resilience, family functioning and peer relationship quality in the model may allow for more theoretically comprehensive analyses. In particular, mediation and moderation models may help clarify the mechanisms underlying the association between nomophobia and social isolation and identify the conditions under which this relationship becomes stronger or weaker.

Despite these limitations, this study provides an important starting point for understanding adolescents’ experiences of social connectedness in the digital age. It provides a meaningful basis for future multidimensional research.

## Author Contributions

The author confirms sole responsibility for the study conception and design, data collection, data analysis and interpretation, manuscript drafting, critical revision and final approval of the submitted version.

## Funding

The author has nothing to report.

## Conflicts of Interest

The author declares no conflicts of interest.

## Data Availability

The data supporting the findings of this study are available from the corresponding author upon reasonable request.

## 1

**TABLE A1:** Group differences in nomophobia scores across demographic and behavioural variables (*N* = 465).

Variable	Group	N	Mean	Std. deviation	Std. error	t	Sig. (two‐tailed)
**Independent samples test**							
Gender	Girl	238	2.8989	0.76438	0.04955	−8.583	**0.000**
	Boy	227	2.3068	0.72121	0.04787		

Variable	Group	*N*	Mean	Std. deviation	df	*F*	Sig.
**ANOVA**							
Age	13	99	2.5879	0.81416	4 460	0.492	**0.741**
	14	90	2.6061	0.90634			
	15	95	2.5284	0.75303			
	16	91	2.6709	0.81612			
	17	90	2.6622	0.70453			
Grade	7	163	2.6052	0.84427	4 460	2.267	**0.061**
	8	31	2.4597	0.87962			
	9	78	2.7051	0.88044			
	10	106	2.4642	0.69545			
	11	87	2.7644	0.69771			
Participation in social activities	1 year or less	26	2.5115	0.73869	3 461	2.047	**0.107**
	2–3 years	58	2.4552	0.83591			
	4–5 years	117	2.5338	0.80932			
	+5	264	2.6873	0.78825			
Daily smartphone use duration (h)	Less than 1 h	13	2.5538	0.72556	3 460	9.648	**0.000**
	1–3 h	127	2.3043	0.79933			
	4–6 h	193	2,6951	0.81719			
	7+	131	2.7901	0.70114			
Time to notice smartphone absence	Immediately notice	206	2.6655	0.79248	3 461	4.443	**0.004**
	Within 10–30 min	221	2.6115	0.79320			
	Within 1–2 h	30	2.4667	0.76748			
	After a longer time	8	1.6687	0.77226			
Emotional response when smartphone is not present	I feel calm	193	2.2376	0.69778	3 461	31.452	**0.000**
	I feel slightly anxious	165	2.8406	0.71206			
	I feel anxious	74	2.7932	0.78406			
	I panic	33	3.2227	0.88079			

Bold values indicate statistically significant results.

**TABLE A2:** Group differences in social isolation scores across demographic and behavioural variables (*N* = 465).

Variable	Group	*N*	Mean	Std. deviation	Std. error	*t*	Sig. (two‐tailed)
**Independent samples test**							
Gender	Girl	238	1.9004	0.77041	0.04994	−3.459	**0.001**
	Boy	227	1.6567	0.74748	0.04961		

Variable	Group	*N*	Mean	Std. deviation	df	*F*	Sig.
**ANOVA**							
Age	13	99	1.7078	0.76504	4 460	0.425	0**.791**
	14	90	1.7500	0.77541			
	15	95	1.8105	0.71328			
	16	91	1.8265	0.83082			
	17	90	1.8175	0.76446			
Grade	7	163	1.7007	0.69699	4 460	1.168	0**.324**
	8	31	1.8203	0.89326			
	9	78	1.8159	0.85175			
	10	106	1.7608	0.69868			
	11	87	1.9130	0.84448			
Participation in social activities	Always participate	149	1.7857	0.78025	2 462	0.039	0**.961**
	Sometimes participate	227	1.7867	0.72150			
	Never participate	89	1.7608	0.86630			
Number of close friends	None	18	2.0317	0.72522	3 461	5.212	0**.002**
	1–2 friends	37	2.0058	0.98693			
	3–5 friends	123	1.9286	0.84812			
	6 or more friends	287	1.6737	0.68218			
Primary communication platform with friends	Face‐to‐face	339	1.7223	0.73008	4 460	4.061	0**.003**
	WhatsApp	70	1.7724	0.64799			
	Instagram	43	2.1960	1.12119			
	Discord	12	2.0298	0.48297			
	Telegram	1	1.6429				
Family attitudes toward internet and social media use	They actively monitor and restrict usage	47	1.8298	0.84299	3 461	0.260	0**.854**
	They warn about risks but do not restrict	276	1.7943	0.77238			
	They do not intervene	107	1.7530	0.74407			
	We never talk about these topics	35	1.7020	0.72647			

Bold values indicate statistically significant results.
